# Mr. Ring's Cases of Secondary Small-Pox

**Published:** 1806-05

**Authors:** John Ring

**Affiliations:** New Street, Hanover Square


### *To the Editors of the Medical and Phyſical Journal.*

GENTLEMEN,

A Compoſition which I communicated to the Royal College of Phyſicians, when they reviſed their Diſpenſatory, about twenty years ago, and which they did me the honour to adopt, is now come into general uſe; and conſidered by many practitioners, as one of the beſt active cathartics and anthelminthics: I therefore apprehend, that a few remarks on this compoſition may not be unacceptable to your readers.

I have now uſed it above thirty years; and experienced the ſuperior advantage of it in many hundreds of caſes, on account of its activity, and the great eaſe with which it is given to children. This latter circumſtance is of no ſmall moment, when we conſider how delicate their ſtomachs are, even in a ſtate of health; and how much their ſufferings are ſometimes aggravated by a nauſeating drug.

In the ſpecimen publiſhed by the College, and ſubmitted by them to the peruſal of medical practitioners, the old formula, called Pulvis Baſilicus, was inſerted; and propoſed to be reſtored to the Pharmacopœia, where it formerly held a place. Having been favoured with the peruſal of the ſpecimen, I drew up a few remarks on this compoſition, and preſented them to Sir George Baker, then Preſident of the College, who communicated them to the Committee appointed to reviſe their Pharmacopœia. I had afterwards the ſatisfaction to learn from him, that my reaſoning on the ſubject met with the approbation of the Committee; and particularly of that ornament of the College, and of the medical profeſſion, the late Dr. Heberden.

I ſtated my opinion, that ſcammony is not a drug which requires a corrector, as had been generally thought; and that if any ſuch corrector were neceſſary, the mode in which it is applied in the pul. baſilicus would ſtill be objectionable. The formula conſiſts of equal parts of ſcammony, calomel, cryſtals of tartar, and waſhed calx of antimony. If an acid is requiſite, in order to correct ſcammony, the abſorbent is detrimental; and if an abſorbent is requiſite, the acid is detrimental. It appears to me, that the calx of antimony and cryſtals of tartar, neutraliſing each other, while they add to the bulk, render the compoſition leſs efficacious, and much more unpleaſant.

It

It is usual to give a powder of this kind in honey or currant jelly; and there can be no other objection to such a mode of administering it, but the difficulty. I have generally directed it to be mixed with a small quantity of water in a spoon, or to be made into a draught; and have seldom been disappointed in the operation. Two drachms of water are sufficient for this purpose; and the calomel in this composition is so well combined with the scammony, by means of the sugar, that very little of it will be lost, provided the phial be properly shaken.

In the basilic powder, the calomel used to subside, even in the bottle where it was kept in a dry form; an objection to which the present formula is less liable; and on that account it has met with the approbation of some of the first characters of the present age.

When I first altered the basilic powder into this form, it was for the sake of giving it with more ease to children who were under preparation for the small-pox; and also after that disease. The necessity of such a medicine, if it ever existed, is now in a great measure superseded by vaccination. But it has other uses; and it has been found practicable to administer this medicine in cases, where a composition of calomel and jalap could not be given, on account of its unpleasant flavour.

These two articles constitute the basis of many nostrums for worms, by which the public are so much deluded. The medicine under consideration is at least equally efficacious, and less disgusting; I have, however, frequently known injections of aloes prove successful in the cure of ascarides, when medicines administered by the mouth had failed; and am inclined to believe, that it is a mode of cure which ought in general to be preferred, because it does not carry off the nourishment contained in the stomach and intestines, nor do so much injury to the constitution in other respects, as violent cathartics exhibited in the usual form.

---

*Cases of the Small-pox a second Time.*

Many false alarms have lately been propagated, in consequence of local small-pox excited in those who had previously undergone vaccination; especially when such local small-pox was attended with fever and other constitutional symptoms. On this account I have already communicated several instances, where the same circumstances occurred in those who had undergone the small-pox; and now add two other cases to the number.

Mrs.

Mrs. Frankham, No 1, Mount Pleasant, Gray's Inn Lane, had the small-pox when young, and is considerably marked with it. Since that time she suckled a child labouring under the disease; in consequence of which, she had several pustules from the contact of matter, which went through the regular course; and were attended with head-ach, fever, and all the other signs of constitutional indisposition, to a considerable degree, for many days.

Mrs. Dean, No. 21, Park Street, Grosvenor Square, was inoculated for the small-pox. Her arm rose; and she had many perfect pustules, attended with the usual constitutional affection. Since that period, she had a number of pustules on her chin, and other parts, by contact of matter from a child at her breast. These pustules regularly maturated, and turned on the 9th day, leaving very large and deep pits behind. She had also the symptomatic fever, and other signs of constitutional indisposition to so great a degree, that the late Mr. Whale, of Oxford-street, who attended her, could scarcely believe she had had the disorder before. This, however, there could be no reason to doubt; had it been otherwise, the pustules, on the last occasion, would not have been merely local.

A case of the small-pox after variolous inoculation, similar to that in the family of the Earl of Westmeath, occurred in a daughter of Mr. Rowan Hamilton, who was inoculated in Ireland, in the year 1787, by Dr. Daniel, a regular physician; and supposed to have the disorder in the most complete and perfect manner. In the month of November, 1806, she caught the small-pox from her brother; and apparently had it a second time.—This case was communicated by Mr. Rowan Hamilton to Mr. Murray, Secretary to the Royal Jenerian Society.

Burserius says, vol. 3, "They also entertain an erroneous opinion, who think, that after once having the genuine small-pox, the disposition of the body to receive that disorder is destroyed; for it appears from undoubted facts, and the investigations of medical men of unquestionable authority, that not a few persons, after experiencing the complaint in the natural way, or by inoculation, have afterwards been affected a second, and even a third time. Hence it is evident, that this disposition is not always destroyed, after once having the disease; or at least, that it may sometimes be excited again."

The following cases were communicated to Dr. Walker by Dr. Huggan, in a letter dated Wallingford, Berks, Jan.

Jan. 2, 1806. They were related to him by an intelligent woman, who was an eye witness to them.

——— Woodley, a native of Harwell, in that county, where his mother still lives, had the small-pox in London, when he was about 25 years old. His face and hands were covered with large broad pustules full of matter. Fifteen years afterwards he was again attacked with the small-pox, when settled at Reading; of which he died. His brother, who went from Harwell to see him, brought back the infection; which rendered a general inoculation necessary at that place.

The woman from whom Dr. Huggan received this information, resided in London about thirty years ago, and was inoculated at the Small-pox Hospital, together with another woman, of the name of Mary Pink, a native of Lewes. They both had the disorder in the regular way; with pustules on different parts, which maturated and turned in the usual manner. About a fortnight after they were dismissed from the hospital, Mary Pink was again attacked with fever, followed by an eruption of the small-pox. She was blind several days; but at length recovered. Another woman living in the next parish to Harwell, was a witness to this circumstance; and recollects it perfectly well. Hence, Dr. Huggan justly observes, it is evident, that the inoculation of the small-pox is no more infallible than that of the cow-pock.

A daughter of the woman who communicated the foregoing cases, slept with her sister who had the small-pox, during the whole course of the disorder; in consequence of which she had two pustules on her knee. She was therefore considered secure from future infection; but three years afterwards she again had the small-pox of the confluent kind; and narrowly escaped with her life.—When she had the local pustules, she laboured under a sore throat, attended with fever; which probably rendered her for a time insusceptible of the constitutional affection of the small-pox; and it is no wonder, that the same thing should now and then happen in the cow-pock.

The following is a case, which I lately received from Mr. Merriman, in a letter dated Jan. 20, 1806; and was communicated to him by Mrs. Dawson, of Shepherd's Market. It occurred to an elderly woman, of Hanslope, in Buckinghamshire, to whom Mrs. Dawson formerly went to school. She perfectly recollects having had the small-pox when 17 years old; which was so severe, as not only to produce pits, but even seams in the face.—Three years years ago she again caught the distemper, when it was epidemic in the town; and had it in so violent a manner, that her life was despaired of. Mr. Dawson's brother, a surgeon at Hanslope, attended her in this second attack; and Mrs. Dawson herself saw her during the eruption.

Another instance of this kind was communicated by Capt. Mowat of Eastbourne; by whose desire the following letter was since transmitted to me by Mr. Verral, surgeon, of Seaford.

"Sir,

"I write to you at the request of Capt. Mowat, to relate the case of Mr. John Wilson, Jun. of Bletchington, whom I attended some time since with the natural small-pox; which he affirms to be the second time of his having that disease. Mr. Pierpoint of Linfield, who inoculated him, is dead; but I have written to Mr. Ward, a practitioner in the same place, who formerly lived with Mr. Linfield as an assistant. This day I had an answer from him, in which he says, that from Mr. Pierpoint's books it appears, Mr. Wilson was inoculated, with two other persons, on the 17th of April, 1783; and left the house on the 7th of May. Mr. Ward recollects nothing of the case; but makes no doubt that the usual appearances took place; and that Mr. Pierpoint was satisfied of his security.

"Mr. Wilson's own account is much more decisive. He says there were above thirty patients with him in the pest-house; he was ill, had a great number of pustules in all parts of the body, and did not recover in less than three or four weeks. He has since been frequently amongst the small-pox; and seen the eruption of that disorder; which he declares to be similar to that which he had in consequence of inoculation.

"In April, 1804, some soldiers of the regiment in Bletchington barracks had the small-pox; and Mr. Wilson, having business there, was much exposed to the infection. On Saturday the 28th of that month, after a day or two of previous indisposition, an eruption began to appear, which he thought resembled the variolous eruption he had seen; but his mother, apprehensive that if he raised an alarm of that kind, it might deter customers from their shop, prevailed on him to confine himself to his room; and no medical man was consulted till the Wednesday following, when they sent for me. When I saw him, I had no hesitation in pronouncing that he had the small-pox; and its progress was from that time so regular, as not to leave a probability of my being in an error. "The

"The nature of the disorder was, however, afterwards proved in a remarkable manner. His mother, with whom he lived, had never had the small.pox. As I was at that time vaccinating many patients in the village, I advised her to submit to that operation; but this she refused to do, and wished me to inoculate her from her son. This, in my turn, I stedfastly refused; as I prided myself that my hands had never been guilty of communicating that disease. We parted, both obstinately adhering to our resolution; but when I visited her son in the evening, I had made up my mind to yield to her prejudices, rather than leave her any longer exposed to the danger with which she was threatened. I assumed, however, for a time, the appearance of being unaltered in my determination, and had the satisfaction of converting her to my opinion.

"She was accordingly vaccinated, and her arm rose and inflamed in the usual manner. On the eighth day an eruption appeared, which, if I had never seen a similar one before, would have alarmed me much. It was a variolous eruption; the consequence of exposure to that infection previous to and during the progress of the cow-pock. Two or three pustules on the face and bosom maturated, the rest began to die away a few hours after their appearance; and by the time the areola was fully formed round the vaccine vesicle, they had entirely disappeared. She had a little fever; perhaps rather more than usually attends the simple cases of cow-pock; but she was not ill enough to require the least confinement.

"Mr. Wilson had a considerable degree of fever, and the eruption was copious; but the disease certainly appeared to be lighter, than is usual with the natural small-pox. This may perhaps be considered as a corroborating evidence of the identity of the first disease; which we may suppose to have weakened, but not entirely to have eradicated the variolous disposition.

"If you wish to make any farther inquiries, I beg you will consider me to be one, who thinks nothing a trouble, that may tend to promote the cause of vaccination, of philanthropy, and of truth.

I am, &c.

C. Verral."

To these cases of the small-pox a second time, I have one more to add. It occurred in a young woman who was inoculated at Chelmsford, when seven years of age. She was ill one day, and had about twenty pustules. Many others others were inoculated at the same time; and no doubt was entertained that they all had the small-pox. Five years after, a woman under inoculation came to the house where she was; and she caught the disease a second time, in a much more severe manner than the first; having a confluent eruption, which has left considerable marks behind. I am not permitted to mention the name of this person; but a reference will be given to her, upon application to Mrs. George, No. 23, Great Castle Street, Oxford Market.

These cases appear to me to be well authenticated; but not more so than the two cases of a similar kind, published in your Journal for March, by Mr. Simpson of Skipton; to whom I beg leave to return, by the same medium, my grateful acknowledgements for his valuable communication.

Mr. Moore, in his Reply to the Antivaccinists, informs us, that he is acquainted with a lady, who had the small-pox from inoculation in her infancy; and who has since had a variolous affection six times, from suckling her children successively, when under inoculation. The pustules filled; and were not distinguishable from the ordinary small-pox. "In one sense therefore," says he, "some persons appear to be susceptible of the small-pox, as often as the contagion is powerfully applied. But this secondary species is commonly a slight indisposition: the fever is usually inconsiderable, the pustules few, small, and soon disappear.

"In the multitude of constitutions, as some are endowed with a singular degree of morbid irritability, these are of course violently influenced by a trifling stimulus. But except in very extraordinary instances, the secondary variolous affection is much milder than the primary: consequently it appears, that an essential change has taken place in the habits even of those who catch it; which occasions a feebler action of the poison. In confirmation of this it should be recollected, that these secondary attacks almost always proceed from the natural infection; which is apt to excite a far more malignant disease than inoculation.

"Many gentlemen of high distinction in medicine, and particularly many strenuous friends of vaccination, infer from such cases as have been taken notice of, that some individuals have the small-pox twice. I know not whether they will be satisfied with the distinction I make, between the primary and the secondary species; for which there appears appears to be a real foundation. I feel inclined not entirely to abandon the principle, that the human body is insusceptible of the true virulent small-pox twice, which has been established by millions of examples; especially as the occasional exceptions admit of an explanation conformable to the rule. Upon this subject, however, I am not very tenacious. There is but a shade of difference between my opinion and theirs; and both of us totally reject the notion of the antivaccinists; who positively deny the recurrence of the small-pox in any shape. These gentlemen appear to be enamoured with this loathsome and destructive malady. They will not suffer any reproach, however just, to be thrown out against it; but flatter it, as they would a deformed heiress."

That there is a clear distinction between the primary attack of the small-pox, and that which usually occurs, must be readily admitted; but the cases which I have here stated, and many others on record, are sufficient to prove that a second attack of the small-pox, in its virulent form, is not so very extraordinary as it is generally supposed to be. In confirmation of this, I shall here subjoin a concise account of some of the most remarkable instances that appear to be authentic.

The first was published in the fourth volume of the Memoirs of the Medical Society of London, in a letter from the late Mr. Withers, surgeon, of Newbury. From his account it appears, that Mr. Richard Langford, of West Shefford, in Berkshire, had the small-pox when a month old, at the same time with three others of the family, one of whom died of it. His face bore the most evident marks of the malignity of the distemper, being so remarkably pitted and seamed as to attract the notice of all who saw him, so that no one could entertain a doubt of his having had it in the most violent degree. Supposing himself to be particularly secure from any future infection of the disorder, he used to frequent the houses of the poor people in his parish, in order to attend them, and accommodate them with such necessaries as their cases required.

In the month of May, 1775, Mr. Withers was sent for to see him; and was informed, that about a fortnight before he had been indisposed for two days, from over-heating himself; but recovered, and continued well till the day before Mr. Withers saw him. "He was then seized with chills, pain of his back and head, &c. with a considerable degree of fever." On the fourth and fifth days his fever fever was somewhat abated, and all the other symptoms mitigated. On the sixth he was still better; and Mr. Withers was informed, that early on the morning of the preceding day they had perceived a rash, which they forgot to mention, and which had escaped Mr. Withers's notice, the chamber being very dark. This eruption was not confined to the face, but extended to his arms, breast, and body. It so much resembled the small-pox, that Mr. Withers told the family he should not have hesitated to pronounce it to be this disorder, if it were not notorious that Mr. Langford had already had it.

The next day the eruption was universal. The throat, of which he had complained the day before, was now become more troublesome; and every other symptom so far favoured the idea of the disease being the small-pox, as to induce Mr. Withers to give the most decided opinion of its being so, and to desire that he might have no intercourse with any of his friends who had not had the disorder. This opinion was ridiculed, and little attention was paid to the precaution; nevertheless, the progress of the pustules, the swelling of the face and head, and the smell peculiar to the disease, as well as every other circumstance, still more and more confirmed Mr. Withers in the opinion he had formed.

On the eighth day two physicians, Dr. Collet and Dr. Hulbert, were consulted, both of whom declared the complaint to be the small-pox; but neither Mr. Langford nor his friends could be reconciled to this idea. The consequence of which was, that the advice of the medical practitioners was not followed, and the patient died on the twenty-first day.

Five of the family being seized with the small-pox, one of whom died, the people in the neighbourhood were convinced that the disease was the small-pox. It was thought so extraordinary a case, that the Rector has recorded it in the parish register.

Mrs. Cook, of Kemp's Court, Berwick Street, was well acquainted with Mr. Langford, and with all the particulars of his case. She declares, that previously to the second attack, he was much pitted, scarred, and seamed with the small-pox. The same account has been given to me by others. Mrs. Cook also informs me, that another woman, besides his sister, caught the small-pox of him; and died of that distemper.

This case, if there were no other on record, is, I humbly presume, abundantly sufficient to prove, that the human body is not "insusceptible of the true virulent smallpox pox twice." But other instances of a similar kind are not wanting. One, which I have already noticed in a former communication, is related by Dr. Buchan; who informs us, that he knew a nurse, who had the small-pox before, so infected by lying in the same bed with a child who had a bad kind of the small-pox, that she had not only a great number of pustules which broke out all over her body, but also a malignant fever, which terminated in a number of boils and imposthumes, from which she narrowly escaped with her life.

Another remarkable case of this kind was published in the Medical Journal for May, 1801, by Mr. Purton of Alcester; who observes, that his father, Mr. Bloxham, a man of sound judgement, who had been extablished in a very extensive business forty years, and attended the patient with him through the whole course of the disease, was equally convinced of its real occurrence a second time. Mr. Purton had before heard of a case of the kind; which was well authenticated, and sufficient to stagger the firmest sceptic.

In the present instance, the patient received the infection of the small-pox, each time, in the casual way. In the first attack, her mother caught the disorder from her; and narrowly escaped with her life. This happened twenty-two years before the second attack; and she had the disease very violently, as the marks which remained sufficiently attested. On the 11th of March, 1801, the whole village of Exhall was inoculated for the small-pox; and this young woman was appointed one of their nurses. On the 3d of April, she was attacked with severe rigors, pain in the head and back, sickness, and the other usual symptoms of an approaching fever. The two following days she was extremely ill: the fever increased to an alarming height, attended with delirium through the greater part of the night, previous to the eruption, which appeared early on the 6th of April. It continued to increase for several days. Her throat became sore, with a sense of fulness, frcm the number of pustules covering the fauces; which continued filling till they were completely maturated. The pustules on the face began to turn on the ninth day of the eruption; and those on the other parts of the body a few days after.

If any case can be stronger than those which are specified, it is that which occurred at Arundel, which I communicated to the public a few months ago, through the medium of this Journal. In that case, the patient was twice covered with the small-pox, at the distance of more than sixty years; and twice *apparently* died of this cruel disease.

The case of Mr. Miles, surgeon, of Dursley in Gloucestershire, published by Dr. Jenner in his Inquiry, is also worthy of attention. This gentleman, who had the small-pox by inoculation, and inoculated not less than two thousand persons, at different times, had reason, according to the notion which then prevailed, to think himself secure from the future infection of that disease. But having inserted a little variolous matter into the back of his hand, in order to show how he performed the operation, a pustule arose; and on the eighth day he was seized with all the symptoms of the eruptive fever, in a much more violent degree than when he was inoculated eighteen years before. As a proof that the first inoculation was effectual, he had a great number of pustules. On the tenth day of the second inoculation, he felt a very unpleasant sensation of stiffness and heat on each side of his face; which terminated in three or four pustules, attended with inflammation. The pustules did not maturate.

The two following cases were communicated to me by Dr. Jenner; and published in my Answer to Mr. Goldson. Mr. Fewster of Thornbury, the celebrated inoculator, had the small-pox in his youth, and was exposed to the infection with impunity for forty years; yet, happening to wound his finger with the point of a lancet, charged with variolous matter, the puncture inflamed and suppurated; and he had a considerable number of pustules on his forehead.

Mr. Scott, another surgeon in the same town, had the small-pox by inoculation; which proved to be of the confluent kind. Nevertheless, he caught it again twenty years after, from a patient whom he was attending; and had it with some degree of severity.

The following case is related by Mr. Hickes, in his Observations on a late publication of Dr. Pearson. It took place five or six years ago, at a farm-house at Arlingham, in the county of Gloucester. A boy had been inoculated with variolous matter, and had been covered with eruptions. About a year after, some of the family having the small-pox, he caught the disease again, and had a very heavy burden.

Mr. Dunning published a case, in which there is great reason to believe the small-pox occurred a second time. Elizabeth Everitt nursed people labouring under the smallpox pox thirty years. This woman supposed she had had the disorder when a child; and there is great reason to believe that this opinion was well founded. Yet she caught it at length, and died. Dr. Geach had related a similar case to Mr. Dunning; which happened to a nurse at the Royal Hospital at Plymouth, under his own observation.

The following case was this day communicated to me by Mrs. Mead, No. 23, Addle Hill, Doctor's Commons; who about twenty years ago, lived in the family of Mr. G. Smith, then residing in Clifford Street. A black woman, who was a native of India, and lived in the same family, being attacked with a violent disorder, told Mrs. Mead, she thought she was going to have the small-pox; which she had already had twice; and of which she bore evident marks. She added, that if it proved to be this disease, she should certainly die; that in India they sometimes had it three times, but in that case they always died. Her words were prophetic; for she had the small-pox, which proved fatal.

The following communication, which I lately received, also tends to corroborate the opinion I now entertain, that the small-pox a second time is no uncommon occurrence.

"*Princes Street, Cavendish Square.*

"Sir,

I have sent you four cases, containing a plain narrative of facts. I cheerfully submit them to you, from a conviction that they will be employed, as far as they are capable, for the advancement of truth and science.

I am, &c.

William Royston."

"*Cases of casual Small-pox succeeding to the inoculated Disease, after an Interval of Nineteen Years.*

Sarah and Mary Taylor were inoculated for the small-pox; Sarah when two years, and Mary when four days old. Sarah was inoculated by a Mr. Richardson, who was engaged in practice with, or was a pupil of Sutton. She had but few pustules, and those faded early, but she was pronounced safe. Mary was inoculated by Mr. Robert Muriel, a respectable country practitioner, and the virus which he used was taken from a person who had the disease by inoculation. She went regularly through the distemper, had a considerable crop of pustules, and an indelible mark remains in her arm where the matter was inserted. During a period of nineteen years these young women, perhaps, were not exposed to the small-pox contagion; but at the end of that time, in February 1788, a general inoculation took place in the village where they resided. Sarah was then attacked with the casual disease; the pustules were confluent in her face, but distinct in other parts; she was blind some days. Mary took the disease at the same time; the pustules on her were numerous but distinct, and turned on the second day.

" *Case of Small-pox occurring a second Time.*

Mary Feakins, of Somersham in Huntingdonshire, had the small-pox in the year 1769, the disease being then very general in the neighbourhood. It appeared in her in its severest form, and she suffered extremely. When she was attacked with the complaint she nursed an infant daughter, then six weeks old, who took the small-pox from her mother, had a confluent kind, was much marked, and lost an eye. In the year 1788, when this daughter was nineteen years old, on being exposed to the accumulated contagion of the small-pox, she was again attacked with the disease; and I attended her. This second attack was not of the slight kind which has been observed to occur in nurses. The eruptive fever continued four days; there was considerable inflammation about the fauces; the number of pustules in her face amounted nearly to two hundred, with a proportionate crop over the surface of the body. The pustules on the face arrived at the acme of maturation on the seventh day from their appearance. Swelling in the face closed the eyes; the extremities were tumefied, and some degree of ptyalism took place. The accumulated effluvium to which this girl was exposed occurred from her sleeping in the same room with three younger sisters, who were down with the disease, one with the casual sort, and two by inoculation.

" *Case of Change in Idiosyncracy from an* insusceptible *to a State* susceptible *of Variolous Contagion.*

Mr. T. C. was inoculated for the small-pox with his family; the punctures on his arm assumed a morbid appearance, but he had no febrile symptoms. During the progress of the disease in the family, one or more of those who had pustules on them, constantly slept with Mr. C. From this test he was deemed safe from the impression of variolous contagion, either from the essential change produced by inoculation having silently taken place in him, or or from some peculiarity of idiosyncracy. Twelve years after this period he became exposed to variolous contagion, from the casual disease appearing in his family. He then took the small-pox, had it in its severest form, and nearly lost his life.

"I now beg leave to subjoin a letter which I some time ago received from Mr. Blair; as a supplement to the account I have already given of the case of Jowles. It relates to the publication of Dr. Rowley, who is now no more. But that is no reason, why the misrepresentations which abound in his work should not be corrected. This is an act of justice to the individuals who are concerned; and to the public."

"*Great Russel Street, Feb.* 11, 1806.

"Dear Sir,

I thank you for the opportunity of perusing Mr. Moore's 'Reply to the Anti-Vaccinists.' I observe, that he notices one of the two cases, respecting which you enquired of me, viz. that of a poor boy called Jowles, "whom Dr. Rowley has so cruelly nick-named the Cow-poxed, Ox-faced Boy, and of whom he has published so hideous a caricature." Mr. Moore very justly denominates it a case of scrofula; for it is nothing else, and there is nothing peculiar in it, unless it be that the boy has tumefied eyelids, which produce a staring aspect, and a redness of the tunica conjunctiva. It is a very singular and far-fetched conceit in the learned Doctor to imagine, "that this boy's face seemed to be in a state of transforming, and assuming the visage of a cow." During the months that I have had the treatment of him at the Bloomsbury Dispensary, his visage has improved, and some of his scrofulous complaints are amended.

"The other case, which you enquire after, is that of a young girl, who had been vaccinated by Dr. Thornton; but unfortunately, after having remained several years in good health, she fell down and broke one of her ribs, which occasioned an abscess in the chest, and for which I am now attending her. I do not know precisely what Dr. Rowley has said or written about this case of Empyema; but I learn from Dr. Thornton, that the public have been here likewise deceived as to the real state of the child, and that her present infirmity has been attributed by Dr. Rowley to the Cow-pock Inoculation!

"Probably you asked for my opinion on these *two cases*, in order to undeceive some persons, who had taken up a

 false false idea respecting them. The hints I send, if they be deemed worthy of attention, are very much at your service: for I feel it an incumbent duty to proclaim real facts as they exist, and am not unwilling to pledge my reputation in support of the truth of what I affirm. The events I now relate are no more the effects of Vaccination than the consequence of cutting the navel string at the birth of the two children in question. I am, &c.

W. BLAIR."

I have many other Observations to offer concerning Dr. Rowley's Work; but these I reserve for a future paper.

I am, &c.

*New Street, Hanover Square.* JOHN RING.

